# Endogenous cortisol correlates with performance under pressure on a working memory task in capuchin monkeys

**DOI:** 10.1038/s41598-022-04986-6

**Published:** 2022-01-19

**Authors:** Meghan J. Sosnowski, Marcela E. Benítez, Sarah F. Brosnan

**Affiliations:** 1 Department of Psychology, Georgia State University, P.O. Box 5010, Atlanta, GA 30302-5010 USA; 2 Language Research Center, Georgia State University, Decatur, USA; 3 Department of Anthropology, Emory University, Atlanta, USA; 4 Center for Behavioral Neuroscience, Georgia State University, Atlanta, USA; 5 Neuroscience Institute, Georgia State University, Atlanta, USA

**Keywords:** Cognitive control, Animal behaviour

## Abstract

Humans often experience striking performance deficits when their outcomes are determined by their own performance, colloquially referred to as “choking under pressure.” Physiological stress responses that have been linked to both choking and thriving are well-conserved in primates, but it is unknown whether other primates experience similar effects of pressure. Understanding whether this occurs and, if so, its physiological correlates, will help clarify the evolution and proximate causes of choking in humans. To address this, we trained capuchin monkeys on a computer game that had clearly denoted high- and low-pressure trials, then tested them on trials with the same signals of high pressure, but no difference in task difficulty. Monkeys significantly varied in whether they performed worse or better on high-pressure testing trials and performance improved as monkeys gained experience with performing under pressure. Baseline levels of cortisol were significantly negatively related to performance on high-pressure trials as compared to low-pressure trials. Taken together, this indicates that less experience with pressure may interact with long-term stress to produce choking behavior in early sessions of a task. Our results suggest that performance deficits (or improvements) under pressure are not solely due to human specific factors but are rooted in evolutionarily conserved biological factors.

## Introduction

As any sports fan knows, people sometimes experience notable and atypical performance deficits when an individual’s outcome depends on that performance. These performance deficits, colloquially referred to as “choking under pressure” (hereafter, choking), have been well documented in how humans perform in a range of high-pressure situations, such as competing for an Olympic gold medal or passing a scholastic exam^1,2^, resulting in a growing interest in understanding the psychological factors that lead to choking to better develop interventions to ameliorate this response. However, not all people show evidence of performance deficits under pressure, and indeed, some individuals seem to thrive under high pressure. Importantly, studies of performance under pressure generally assume that choking is a human phenomenon, a consequence of our advanced cognitive abilities coupled with internalized societal pressures. It is possible, however, that choking need not rely on human-specific cognitive traits but is a phylogenetically older response shared by other animals. Stress responses are well conserved across species, raising the possibility that other animals, and in particular, other primates, show similar effects^3^. To date, little research has explored whether non-human species also choke (or thrive) under pressure, yet this has important implications for understanding both the evolution of this behavior and the role of human-specific factors, such as self-awareness and cultural demands, on performance deficits.

In humans, choking under pressure is exemplified by an experienced individual failing to complete a task that they have previously completed successfully when they are under psychological threat, such as time constraints, that are typically experienced as stressful. Quixotically, not all people do choke, and in fact, the reverse can happen, with some individuals thriving under pressure, leading to individual differences in responses within the human literature. Previous studies found that the likelihood of choking depends on a person’s expertise with the task, the specific cognitive demands involved with the task, and the strategies that the person uses to complete the task^4–6^. Further, in situations involving cognitive rather than physical performance, tasks that engage and rely on working memory seem to be particularly sensitive to pressure demands, possibly because working memory requires sustained focus and attention that acute pressure might disrupt^2,7^. Indeed, those who perform the best on working memory tasks under normal pressure conditions are counterintuitively the most likely to choke under high-pressure conditions, perhaps suggesting that they are using more complex working memory strategies that break down more quickly as pressure increases^2,7,8^.

There are three main hypotheses attempting to explain what happens when people fail to perform under pressure. All of them involve the reallocation of attention away from the task at hand, but they differ in predicting to where that attention is shifted. The distraction hypothesis notes that the experience of pressure is uncomfortable and predicts that attention is pulled away from the task to focus on the uncomfortable experience caused by the immediate stress response, which reduces the available cognitive resources needed to complete the task^9^. In contrast, the explicit monitoring hypothesis also suggests that attention is pulled away from the task at hand, but because the person becomes acutely aware of their performance and the actions needed to complete the task, heightening self-consciousness. Counterintuitively, this change in attention impedes performance rather than improving it, which has been shown in a variety of cognitive domains^9,10^. Finally, the over-arousal hypothesis predicts that a highly desirable incentive draws attention away from the task, and that this incentive proves to be too arousing for the individual to focus on performing the task properly. The over-arousal hypothesis is based in the Yerkes-Dodson law, which posits that for a given task there is a state of arousal that is optimal to perform that task, and beyond that optimal point, performance decreases with increasing arousal^11^. In humans, the explanatory value of these three hypotheses can be difficult to tease apart, as they are likely not mutually exclusive (indeed, to some degree predictions overlap) and there is some evidence for all three^12^. However, given that other species may not show evidence of some cognitive capabilities on which these theories hinge (i.e., self-awareness about performance, as in the explicit monitoring hypothesis), studying them is particularly helpful for determining which accounts underlie choking behavior.

It seems likely that stress impacts decision-making in animals in similar ways, and that similar factors might be related to individual responses to pressure in non-humans. Animals face decisions in high-pressure situations that impact their likelihood of survival, such as when facing a predator and deciding whether to fight or to flee, and for which immediate stress responses have a significant impact on behavioral outcomes^13,14^. Therefore, the ability to manage responses to acute stress was presumably selected for. Moreover, studies in other non-human primates have shown evidence of working memory (the system most sensitive to effects of pressure) or a working-memory-like system, and patterns of decision-making in primates are often comparable to those observed in humans^15,16^. Indeed, areas of the brain related to working memory are also impaired by stress in primates^17^, suggesting that individual factors related to choking under pressure in humans might also apply to non-human primate species.

Another factor that may be important in understanding choking is the impact of the individuals’ current state of stress. Long-term, or chronic, stress is well-documented to have important cognitive consequences, resulting in impaired declarative memory and impacts on brain function in areas important for memory, decision-making, and learning^18,19^. What is less well studied is how acute stress caused by pressure in-the-moment might interact with chronic stress to produce the cognitive deficits that are a hallmark of choking. Recently, there has been growing interest in how hormonal profiles might at least partly explain individual differences in cognitive performance. Cortisol, a hormone implicated in the stress response, is an important candidate in this. Beyond interfering with working memory itself, as discussed above, high pressure might also activate the stress response, a distracting experience in itself that adds to the attentional load required to complete the task and decreases an individual’s ability to perform using working memory^8^. Human studies suggest that cortisol levels interact with cognitive traits such as working memory capacity in these tasks, such that high working memory individuals are more likely to fall prey to the choking phenomenon in cognitive tasks with higher increases in cortisol over the course of the task at hand^12,20,21^. Because the stress response has been well-conserved^3^, if animals are also sensitive to pressure, we have reason to expect that cortisol would be related to their responses as well.

**Figure 1:**
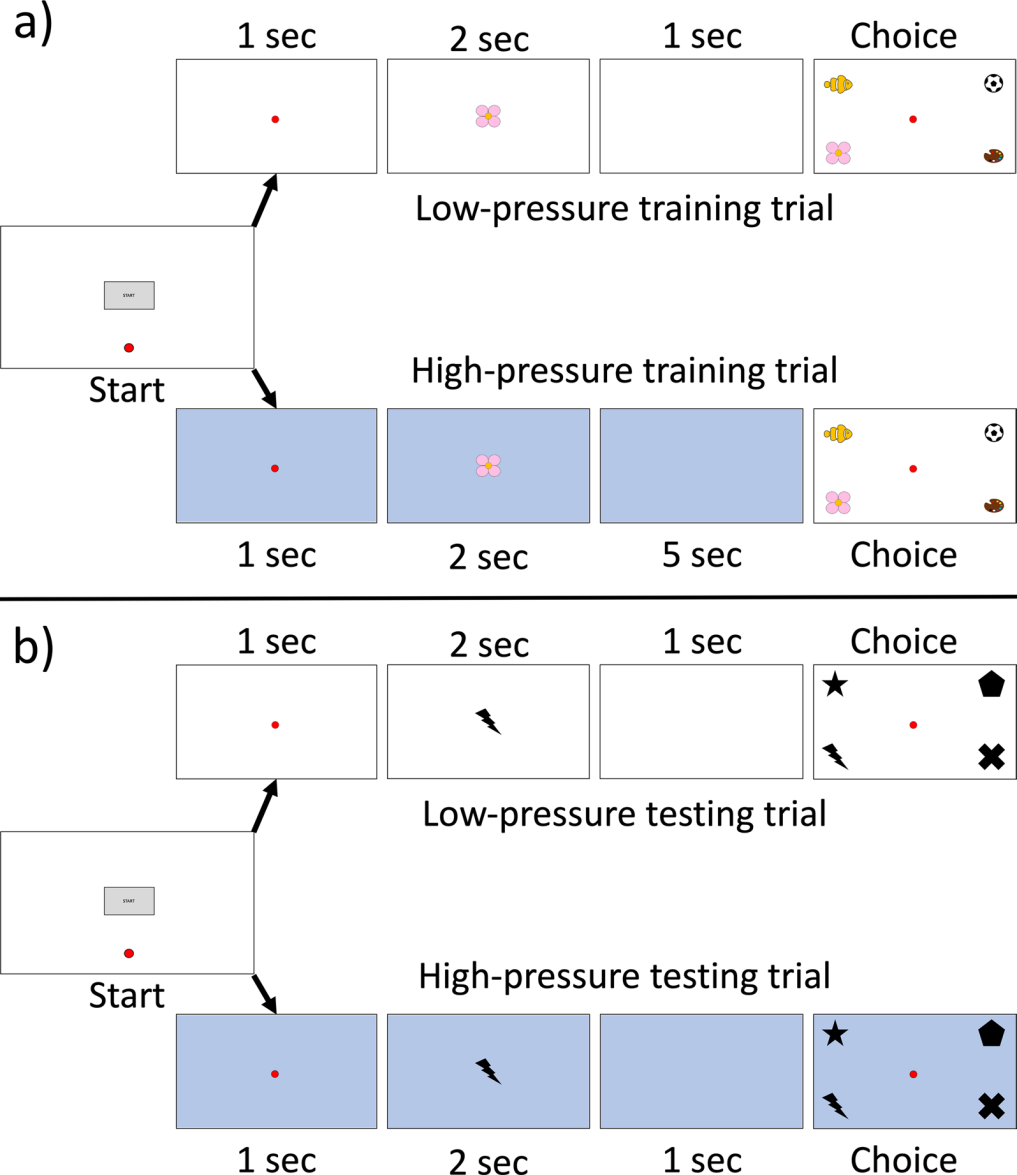
Side-by-side comparison of high-pressure and low-pressure trials in both training (**a**) and testing (**b**). While the trained background color cue occurs in the high-pressure test trial, all delays are the same as in the low-pressure test trial, removing any confounds of higher difficulty.

In this paper, we had two aims: first, to assess whether tufted capuchin monkeys (*Sapajus [Cebus] apella*), like some humans, choked (or thrived) under pressure in a memory task, and second, to determine if, again like humans, monkeys’ cortisol levels were correlated with individual differences in performance under pressure in that task. Capuchins are an ideal species for this study as past research shows that they have cognitive processes that resemble working memory in human subjects^15,22^ and that negative experiences impact both behavior and performance on a subsequent cognitive task (i.e. a match-to-sample task)^23^, suggesting that individuals might also be prone to the cognitive effects of pressure. To that end, we tested subjects from a captive colony of tufted capuchin monkeys that live in species-typical mixed-sex social groups with stable social organization and participate only in non-invasive cognitive and behavioral testing. To test their responses to pressure, monkeys were trained on a computerized matching task in which a subset of trials were accompanied by cues that were previously trained to indicate high-reward, high-pressure trials, interposed among regular (low-pressure) trials, but all with the same level of difficulty (Fig. 1). As these monkeys do not experience significant stressors (i.e., social separation, invasive testing) we would expect that, as with typical humans, even small stressors such as pressure may cause cognitive and behavioral effects.

If subjects are sensitive to pressure, their performance should be different on trials with the high-pressure cues than on low-pressure trials, despite the identical difficulty level between the two types of trials. Given human differences in propensity to choke or thrive and capuchins’ individual differences on working memory tasks^15,24^, we predicted the existence of individual differences in how capuchins responded to high pressure trials. To assess the relationship between individual differences in naturally-occurring cortisol and a given monkey’s change in performance between trial types, we concurrently collected fecal samples throughout the testing period. We predicted that, as with humans, if cortisol was related to how individuals performed under pressure, the performance decrease between low- and high-pressure trials should be larger in individuals with higher cortisol levels.

## Results

### How do capuchin monkeys vary in their ability to perform under pressure?

To assess if capuchin monkeys, like humans, vary in their individual susceptibility to choking under pressure, we conducted a computerized delayed-match-to- sample task (DMTS; see “Methods” section for details) in which some trials were designed to induce a higher level of pressure without an increase in task difficulty. After training the monkeys to associate a blue background with a trial that was both harder (5 s delay between sample presentation and presentation of the matches rather than the typical 1 s) and more rewarding (three rewards instead of one), we tested 20 capuchins (13 female; 7 male) on 15 sessions of up to 200 trials each (trials per session: *M* = 189.57, Range: 22–200) in which “high pressure” trials (25%) were interspersed with regular trials (75%), but without the extra delay (Fig. 1) to isolate the effect of pressure due without confounding it with the effect of difficulty. Only eight novel stimuli were used in testing to ensure that their DTMS response utilized working memory rather than familiarity^25^.

To assess whether, overall, capuchins performed differently on the high-pressure trials in the testing sessions, we built a linear mixed model with proportion of correct trials in that testing session as the dependent variable, the trial type (high- or low-pressure) as a fixed effect, and monkey ID as a random effect. Because capuchins have been shown to improve on their performance on memory tasks over time^26^, we included session number as a fixed effect. In addition, because there is some evidence that there may be a sex difference in how individuals respond to stress generally^27^, and specifically in working memory performance under stress^28^, we included sex as a fixed covariate in our model. We found no overall effect of pressure condition (β = 0.00, 95% CI = [− 0.02, 0.02], *t* = − 0.33, *p* = 0.74) or of sex (β = 0.06, 95% CI = [− 0.01, 0.14], *t* = 1.67, *p* = 0.10) on how capuchins performed on high- and low-pressure trials. However, session number was predictive of performance (β = 0.01, 95% CI = [0.01, 0.01], *t* = 10.34, *p* < 0.001), with capuchins performing better with experience with the task, although the effect size was small (Table 1).

**Table 1:** Linear mixed-model of proportion correct predicted by pressure condition and session number, with subject included as a random effect.

Predictors	Estimates	SE	Conf. int. (95%)	*p*
**Proportion correct**				
(Intercept)	**0.42**	0.02	0.37–0.47	**< 0.001**
Condition	0.00	0.01	− 0.02 to 0.02	0.741
Session number	**0.01**	0.00	0.01–0.01	**< 0.001**
Sex	0.06	0.04	− 0.01 to 0.14	0.095
N_Subject_	20			
Marginal R^2^/conditional R^2^	0.261/0.521			

For the categorical predictor of pressure condition, the intercept is “high-pressure”; for categorical predictor of sex, the intercept is “female”.

Significant estimates and their *p* values are bolded.

### Does cortisol correlate with how individuals perform under pressure?

Despite the fact that pressure condition was not predictive of capuchins’ performance as a group, based on human results^2,7^, we predicted that there would be individual differences in choking that were related to differences in individuals’ baseline cortisol levels. To explore this, we focused on the difference between high- and low-pressure performance (rather than overall memory performance by each individual) by calculating a difference score for each testing session, which was the difference between proportion correct on high-pressure trials and proportion correct on low-pressure trials within that testing session. Thus, negative difference scores indicated worse performance under high pressure, or choking, while positive difference scores indicated better performance under pressure, what we might call thriving. Because the difference score, by definition, included both trial condition types, it provided a single score for each individual that presented how they performed differently on high-pressure trials in the context of how they performed on typical trials within each session.

**Figure 2:**
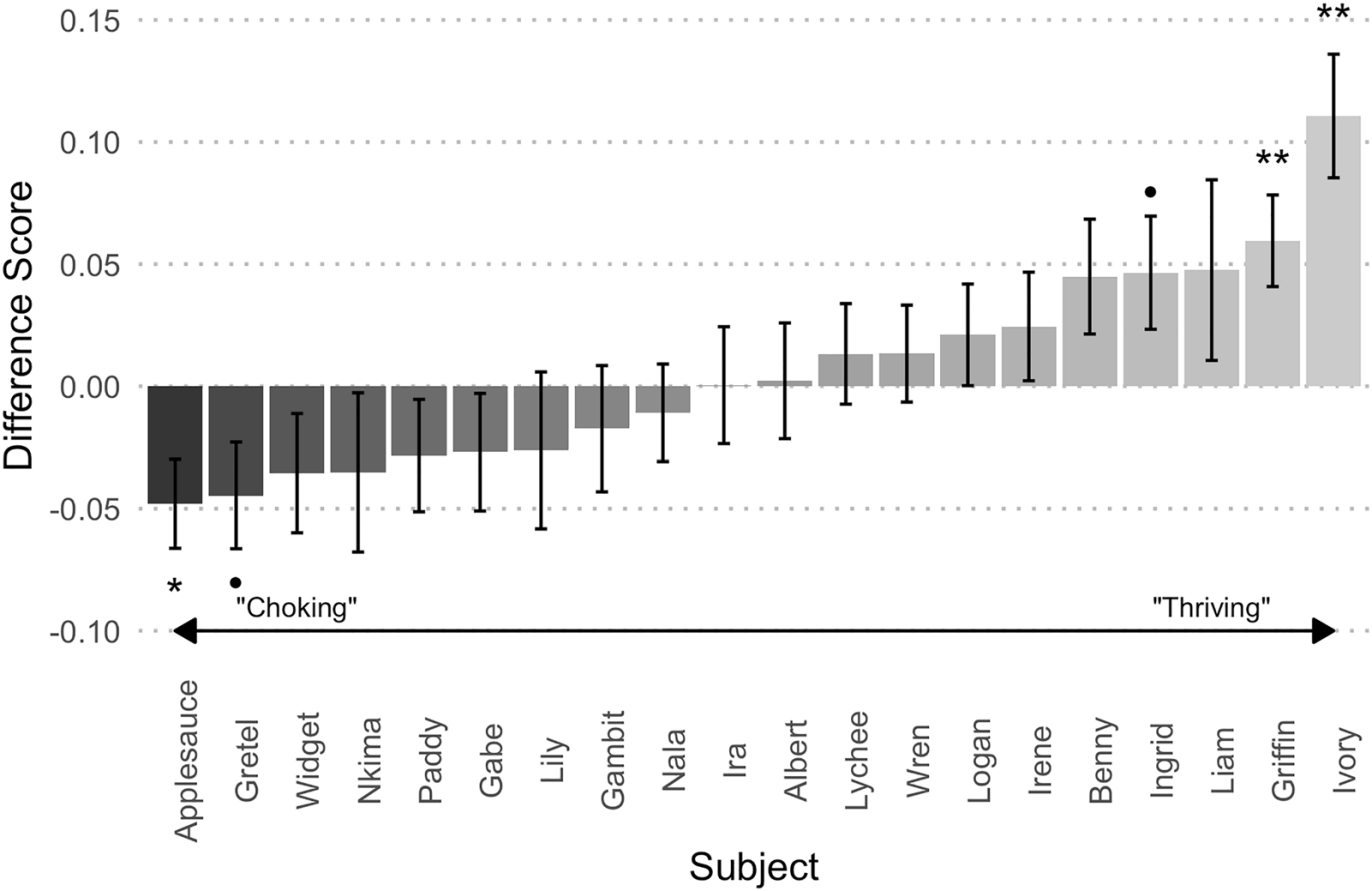
Bar graph of average overall difference score throughout 15 sessions for each individual. Positive scores indicate better performance on high-pressure trials (HPTs) than low-pressure trials (LPTs) (thriving under pressure); negative scores indicate worse performance on HPTs than LPTs (choking under pressure). Error bars represent the standard error of the mean (SE). •*p* < 0.10, *p < 0.05, **p < 0.01; results of non-parametric Wilcoxan signed rank tests.

As predicted, individual monkeys differed significantly in how they responded to pressure (one-way ANOVA of subject on difference score: *F* (19, 280) = 2.85, *p* < 0.001, *η*^2^_*p*_ = 0.16). A visual inspection of the difference in performance between high- and low-pressure trials showed both monkeys who tended towards choking and monkeys who tended to thrive (Fig. 2). To assess whether there was a relationship between endogenous cortisol levels and individual subjects’ performance on the DMTS task, we collected 124 fecal samples from 19 subjects in total (per individual: *M* = 4.59, Range: 1–13; we were unable to obtain a fecal sample from one individual, so the following mixed-modeling analyses are based on 19 subjects rather than 20). We used a commercially available enzyme immunoassay (Arbor Assay) previously validated for use in capuchins to examine cortisol levels (mean inter-assay CV = 26.48%; mean intra-assay CV = 7.45%).

**Figure 3:**
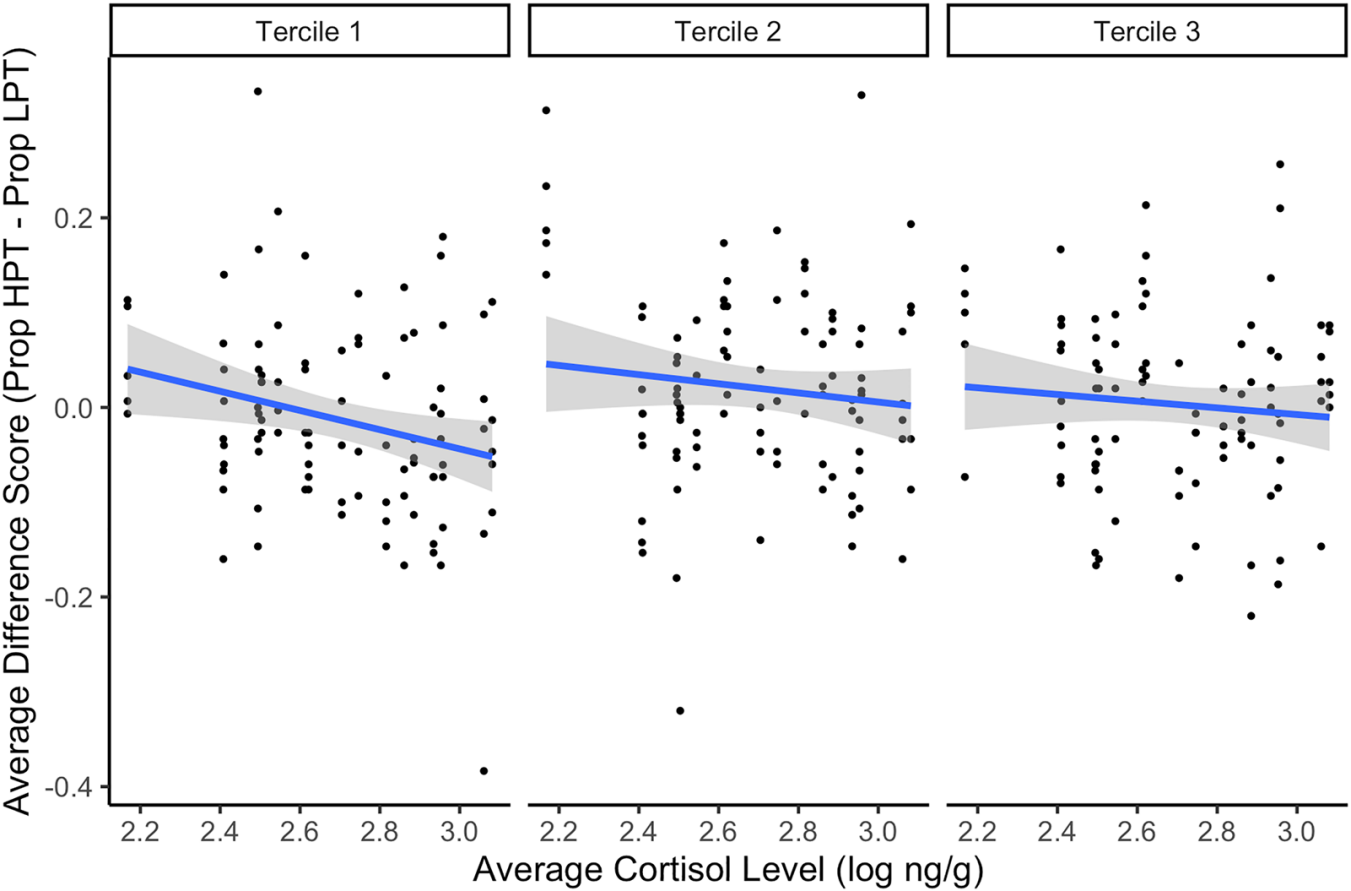
Cortisol’s relationship to performance, based on experience with the task. For visualization purposes, we split session numbers into three terciles (First tercile = sessions 1–5, Second tercile = sessions 6–10, and third tercile = sessions 11–15). Confidence bands represent a 95% confidence interval.

We fit a linear mixed model that statistically predicted difference score from the fixed effects of average cortisol (log-transformed; see “Methods” section) and session number, given our previous finding that session number was related to performance; we also included subject as a random effect term (Table 2). Overall cortisol level was negatively related to difference score (β = − 0.12, 95% CI = [− 0.22, − 0.01], *t* = − 2.16, *p* = 0.03; Fig. 3). However, in this model, neither session number (β = − 0.02, 95% CI = [− 0.05, 0.01], *t* = − 1.22, *p* = 0.22) nor the interaction between average cortisol and session number (β = 0.01, 95% CI = [0.00–0.02], *t* = 1.34, *p* = 0.18) were significantly related to difference score.

**Table 2:** Linear mixed-model of difference score predicted by average cortisol and session number, with subject included as a random effect.

Coefficient	Estimates	SE	Conf. int (95%)	*p*
**Difference scores**				
Intercept	**0.31**	0.15	0.01–0.60	**0.036**
Cortisol (log ng/g)	**− 0.12**	0.05	− 0.22 to − 0.01	**0.031**
Session number	**− **0.02	0.01	− 0.05 to 0.01	0.221
Cortisol × session number	0.01	0.01	0.00–0.02	0.181
N_Subject_	19			
Marginal R^2^/conditional R^2^	0.033/0.112			

Significant estimates and their *p* values are bolded.

## Discussion

Our results suggest that, like humans, there is individual variation in how capuchins perform on a cognitive task during high-pressure situations, with some monkeys tending to choke and others to thrive. While we did not find an overall effect of pressure on cognitive performance, these results were not surprising given previous studies in humans. In humans, not all people choke (in one commonly-cited study assessing penalty kick performance in a professional soccer league, at most only 7.5% of all kicks were missed as a result of choking, defined as failing to shoot on target)^29^ and even in studies that do find an effect, the effect size tends to be small^2,7,30^. Because of this variation, research has focused on unpacking the factors that are related to whether an individual chokes or not. Here, we found that cortisol was correlated with each monkey’s proclivity for choking or thriving behavior such that individuals with higher average cortisol levels performed worse under pressure. In addition, overall performance was weakly related to experience with performing under pressure, supporting prior research that found that practicing under pressure improves later performance under pressure^31^. As monkeys generally improved their performance in later sessions, we propose that early sessions might be more reflective of responses to pressure (the choking phenomenon), while later sessions might have tapped into the effects of practice, learning, and motivation, especially if the monkeys eventually recognized that high-pressure trials were not harder. In addition, the high-pressure trials were inherently more motivating because they continued to reward a higher number of pellets, even in the testing phase, which presumably functioned to increase performance. Although we recognized this possibility, we chose to keep the higher reward to avoid giving the monkeys an extra cue that the high-pressure trials were different in the testing phase, which could have changed performance independently of the effect of pressure. Future work, however, may wish to focus on fewer trials and sessions to catch performance prior to monkeys’ recognition of this change. In addition, other sources of individual variation might be explored, such as ongoing social dynamics or measures of personality traits.

We found evidence to support a relationship between fecal cortisol levels and choking behavior, which suggests that ongoing exposure to stress is related to the ability of an individual to cope with an acutely stressful situation and, therefore, the individual differences we see in choking. Although we cannot establish a causal or predictive link because we assessed endogenous cortisol in the absence of a hormonal manipulation, this fits with the previous understanding of stress’ effect on cognition. Chronic stress alone affects cognitive performance, impairing memory performance for neutral stimuli like the abstract shapes that we used in our study^32^. In this study, we examined average fecal cortisol, as a more robust measure of long-term stress than other noninvasive methods, like saliva and urine. In capuchins, fecal cortisol represents an average measure of excreted cortisol for up to eight hours prior to collection^33^. For most subjects, we were able to collect multiple fecal samples throughout the study period, except for four subjects for whom we had only one fecal sample. It is possible that the cortisol levels for those four individuals are not indicative of their chronic stress, however, even with this potential confound, we found evidence of a significant relationship between fecal cortisol and performance. Future studies would benefit from assessing the difference between current stress, on the day of testing, versus long-term chronic stress.

Chronic stress might also interact with in-the-moment stress responses to produce behavioral reactions; in previous literature, chronically stressed individuals showed a suppressed cortisol response to new, acute stressors^34^, but showed increased behavioral reactivity to those stressors^35^. Although we did not assess the short-term changes in cortisol in this study, we predict that future work will find that behavioral outcomes under pressure may be the result of an interplay between chronic stress state and immediate stress responses, such as that assessed using changes in salivary cortisol as a measure of immediate stress response^33^. In order to explore the behavioral outcomes of this relationship, future studies could include behavioral measures of arousal and stress (for instance, video coding of body movement and vocalizations during the task, eye tracking ocular saccades during high-pressure trials, measuring heart rate variability, or measuring latency to complete a trial); these methods may provide an important manipulation check to verify that the task is, in fact, inducing pressure. Although acute stress is not itself synonymous with pressure (by our definition, the former would be a consequence of the latter), a measure of in-the-moment stress would provide further evidence of an interaction between chronic stress and immediate stress responses.

We chose to use the DMTS task because it has been used to assess working memory in capuchin monkeys previously^15^, and choking may be particularly prevalent in working memory tasks in both humans and other species. However, in the DMTS task, subjects do not need to manipulate the information being remembered, suggesting it may involve short-term declarative, rather than working, memory^2,36,37^. We mitigated this in our task by using small set of stimuli in our testing sessions, which has been shown to separate the effects of active working memory from familiarity effects^25^. Nonetheless, future work should explore tasks that use other methods for involving working memory to ensure that these results generalize. Additionally, future work might explore how working memory capacity is related to^14^ performance under pressure, as shown in humans^20^.

While choking in humans can bring to mind epic failures, studies of choking in human participants often focus on small changes in performance under pressure in a subset of subjects, as we found with our monkeys. In our study, the difference in performance on the high-pressure trials compared to low-pressure trials was modest, up to a 5% decrement or 10% increase overall, suggesting that it is in the range of human responses in such studies^2,7,30^. However, small impacts build up, even in low-stake situations, and a decrease in performance of 5% in life-or-death situations could have fatal consequences. Higher evolutionary stakes than in our task might induce more pressure and in turn interfere with working memory more, as cognitive resources are reallocated to processes more critical for survival. Moreover, more monkeys may be susceptible to pressure effects, and subjects’ baseline cortisol (i.e., levels of chronic stress) might matter even more than in the low-stakes situation that our study tested. In addition, sometimes high-pressure can be motivating, as evidenced that some of our capuchins performed better on the high-pressure trials overall; this makes sense, as better performance under pressure would lead to better outcomes in ecologically relevant high-pressure situations (for instance, facing a predator).

Our study contributes to the growing literature on how a high-pressure situation affects cognitive performance by specifically examining how non-human animals perform in response to pressure. The fact that we see some evidence for choking in other species suggests that humans’ responses are not due to humans’ language^38^, high-level cumulative cultural evolution^39^, or well-developed theory of mind^40^, but factors shared more broadly across animals. Moreover, because we saw choking in a species that does not show evidence of self-consciousness about their performance (or indeed, evidence of self-awareness in general^41^), our results support a distraction or over-arousal account of choking, rather than the one proposed in the explicit monitoring hypothesis. This is not to say that self-consciousness does not play a role in human choking, but instead suggests that the evolution of choking was more likely related to reallocated attention as a result of the stress response. Specifically, our data suggest that in monkeys, as in humans, cortisol, a naturally-occurring stress hormone, correlates with differences in cognitive performance under high pressure. Our data support a model of individual differences in choking under pressure in which an individual’s long-term cortisol level is negatively correlated with performance under pressure in early attempts to perform, but experience with performing under pressure mitigates these negative effects. Future studies would benefit from examining how long-term cortisol levels interact with acute cortisol response to further influence (or mitigate) differences in response to high pressure in the context of this model and how these effects vary in higher-stakes situations. In the long term, understanding these effects in other species will help us better predict and ameliorate these effects in humans.

## Methods

### Subjects and testing apparatus

Subjects consisted of 20 tufted capuchin monkeys (7 male, 13 female; age range: 7–44 years) housed at the Language Research Center (LRC) of Georgia State University (GSU). All monkeys have *ad-libitum* access to running water at all times, including during testing. Subjects were previously trained using positive reinforcement to voluntarily enter individual testing chambers (0.60 mL × 0.35 mW × 0.45 mH) attached to the indoor section of their home enclosures, allowing them to participate in cognitive testing while maintaining visual and vocal contact with groupmates. Monkeys typically had this opportunity seven days a week, if they so chose, making this a normal part of their routine. Each group also has daily access to an outdoor enclosure; for more information about indoor and outdoor enclosures, please see Table S4 in Supplementary Information. Subjects were never deprived of food, water, or outdoor or social access to encourage testing, and there were no consequences to failing to come into the test enclosures other than not being able to participate in the task. For more information about subjects and testing setup, please see Supplementary Methods online.

Once monkeys were separated, they were given access to their individual computer testing apparatus. Subjects had been previously trained to use a modified joystick controller to control a cursor on a computer set-up to participate in cognitive tasks^42,43^. Throughout the experiments, they were automatically rewarded for correct choices or for certain decisions via an attached pellet dispenser that released banana flavored 45 mg Bioserv pellets. Testing sessions typically lasted between one and two hours, the length of which was determined by how quickly subjects completed the trials.

### General procedure

We designed a delayed match-to-sample (DMTS) computer task in which most trials were low-pressure trials typical of a normal task and resulting in a typical reward, but some trials were high-pressure trials, with cues that had been trained to denote a harder trial, but one that resulted in a better reward for a correct response. To do so, we first trained the monkeys to associate a blue background with a harder DMTS trial consisting of a longer delay^26,44^ (5 s) and resulting in a reward of three pellets, compared to a low-pressure trial, with a one second delay and resulting in one pellet. In testing, we used an unfamiliar set of stimuli and kept the cues the same while removing the delay difference between trial types, thus equalizing difficulty while retaining pressure cues. A schematic of high- and low-pressure trials in both training and testing are depicted in Fig. 1.

### Training

All monkeys were previously familiar with the DMTS task, so training was designed to teach them that a blue background indicated a harder trial while a white background indicated an easier one. Training was done using ClipArt stimuli with which the monkeys were familiar from previous studies, and subjects completed as many trials as they could in a typical daily session (up to 5 h, which is a typical training day for this population; trials per session: *M* = 436, *SD* = 248, Range: 7–1512) with one in every four trials being a high-pressure trial, randomized within that block of four. To start a trial, the subject moved the cursor to a “start” button. If the trial was high-pressure, the background of the screen turned blue; otherwise, the background of the screen remained white. Then, a sample image was presented in the center of the screen for two seconds, after which it disappeared. If the trial was high-pressure, there was a delay of five seconds; if the trial was low-pressure, the delay was one second. After the delay, four choice images were displayed, one in each corner of the screen; one of these images matched the previously shown sample. If the monkey selected the correct image, they were rewarded with three pellets for a high-pressure trial or one pellet for a low-pressure trial; although it might seem a small difference in reward quantity, we have evidence that this population of capuchins is sensitive to the number of reward pellets received and changes their behavior accordingly^45^. After an inter-trial interval of two seconds, the start button reappeared to start a new trial. Thus, in training, high-pressure trials consisted of a different cue, a different difficulty, and a different reward. All subjects completed trials in the training phase until they were able to score least 75% correct on both high- and low-pressure trials within a given session, which ensured that the monkeys were performing well above chance (25% for a four-choice task). This also ensured a comparable level of competency across subjects, as well as room to drop in performance while still being above chance.

### Testing

After each subject reached criterion, they were moved to the testing phase. For testing trials we used a set of eight unfamiliar and unique stimuli, rather than the familiar ClipArt (see Fig. 1), and we removed the longer delay in high-pressure trials in order to isolate the effect of pressure from the effect of difficulty. To minimize the chance that subjects would stop responding to high-pressure trials differently than low-pressure trials, subjects were constrained to 200 trials per session and two sessions per week. As in training, within each session we set a trial ratio such that one in every four trials was a high-pressure trial, so subjects completed 150 low-pressure trials and 50 high-pressure trials. Subjects completed 15 sessions of the task in the testing phase. The program automatically recorded subject, date, time, pressure condition, response, and all stimuli used for each trial completed by the subject.

### Hormone sampling

To assess cortisol from each subject, we used a non-invasive fecal sample collection method. We collected fecal samples opportunistically from beneath each subject’s testing box between 8:30 AM and 11:00 AM to minimize the daily fluctuations in cortisol levels as a result of diurnal rhythm. We collected fecal samples as often as available, at minimum once every seven days per monkey. At time of collection, we recorded individual, date, and time. Fecal samples were then frozen at − 20 °C until time of elution at the LRC.

We thawed the samples and eluted them following protocols found in previous literature^33^, using 80% ethanol into 2.0 mL microcentrifuge tubes, after which samples were refrozen for transport to the Neuroscience Core Facility at the GSU main campus. Samples were dried down, then reconstituted and analyzed using a commercially available enzyme-linked immunoabsorbent assay (ELISA) assay kit (Arbor Assays, Ann Arbor, MI) that we validated for use with capuchin fecal samples (see Supplementary Data for linearity validation).

### Statistical analysis

We first focused our models on understanding the effect of the different levels of pressure alone, as well how that pressure might interact with experience with the task. We built a linear mixed model (LMM) that predicted the proportion of trials correct in each session from the fixed effects of pressure condition and session number, including subject ID as a random effect in the model. We also included sex as a fixed effect, but as sex did not significantly affect performance, we removed it from later models (see “Results” section).

Given the presence of individual differences in human subjects, we predicted that monkeys might vary as a result of some individual factor. To test this, we first calculated an outcome variable of a difference score that represented the difference in performance across the two conditions. We first conducted a one-way ANOVA to assess if there were individual differences in difference score among the subjects; we also created and visually inspected a bar plot of average difference score by individual. Both of these measures indicated that there was reason to explore individual factors that could be related to performance.

To that end, we assessed how hormonal profiles might be related to individual differences in how subjects reacted to pressure condition by analyzing individual levels of cortisol. We used difference score as the outcome variable for a linear mixed-model analysis. For cortisol, we averaged cortisol levels for each individual to create a “baseline”, which is more representative of chronic stress. We used log-transformed cortisol scores due to non-normality of the raw cortisol values, which is a common correction for non-normality of hormonal data in previous literature^46^. Using these variables, we built a model that predicted difference score from the fixed effects of average cortisol, session number, and the interaction between the two; we also included subject ID as a random effect in this model.

All statistical analyses were run using the base, *lme4*, *lmtest*, and *glmer* packages of R in RStudio^47,48^. All outcome variables and predictor variables were checked for normality and relevant assumptions of linear models prior to analysis; for instance, we visually inspected Q-Q plots of the residuals of outcome variables to ensure that they were normally distributed. For each model that we ran, we conducted a likelihood ratio test to compare that model to the null model, which included only the intercept with random effects.

### Ethics statement

All protocols involved in this study were approved by the Georgia State University IACUC (#A19027). Additionally, all protocols and procedures used in this study complied with the relevant legal requirements governing animal research in the United States of America. The project reporting complied with ARRIVE guidelines.

## Supplementary Information

Supplementary Information.

## Data Availability

The data that support the findings of this study are available from the corresponding author upon reasonable request.

## References

[1] Hill DM, Hanton S, Fleming S, Matthews N (2009). A re-examination of choking in sport. Eur. J. Sport Sci..

[2] Beilock SL, Carr TH (2005). When high-powered people fail—Working memory and “choking under pressure’’ in math. Psychol. Sci..

[3] Schoenle LA, Zimmer C, Miller ET, Vitousek MN (2021). Does variation in glucocorticoid concentrations predict fitness? A phylogenetic meta-analysis. Gen. Comp. Endocrinol..

[4] Baumeister RF (1984). Choking under pressure: Self-consciousness and paradoxical effects of incentives on skillful performance. J. Pers. Soc. Psychol..

[5] Lewis BP, Linder DE (1997). Thinking about choking? Attentional processes and paradoxical performance. Pers. Soc. Psychol. Bull..

[6] Beilock SL, Carr TH (2001). On the fragility of skilled performance: What governs choking under pressure?. J. Exp. Psychol. Gen..

[7] Gimmig D, Huguet P, Caverni J-P, Cury F (2006). Choking under pressure and working memory capacity: When performance pressure reduces fluid intelligence. Psychon. Bull. Rev..

[8] Beilock SL, DeCaro MS (2007). From poor performance to success under stress: Working memory, strategy selection, and mathematical problem solving under pressure. J. Exp. Psychol. Learn. Mem. Cogn..

[9] Wine J (1971). Test anxiety and direction of attention. Psychol. Bull..

[10] Lyons IM, Beilock SL (2012). Mathematics anxiety: Separating the math from the anxiety. Cereb. Cortex.

[11] Yerkes RM, Dodson JD (1908). The relation of strength of stimulus to rapidity of habit-formation. J. Comp. Neurol. Psychol..

[12] Yu RJ (2015). Choking under pressure: The neuropsychological mechanisms of incentive-induced performance decrements. Front. Behav. Neurosci..

[13] Li L (2018). Stress accelerates defensive responses to looming in mice and involves a locus coeruleus-superior colliculus projection. Curr. Biol..

[14] Voellmy IK, Goncalves IB, Barrette M-F, Monfort SL, Manser MB (2014). Mean fecal glucocorticoid metabolites are associated with vigilance, whereas immediate cortisol levels better reflect acute anti-predator responses in meerkats. Horm. Behav..

[15] Tavares MCH, Tomaz C (2002). Working memory in capuchin monkeys (*Cebus apella*). Behav. Brain Res..

[16] Beran MJ, Parrish AE (2012). Sequential responding and planning in capuchin monkeys (*Cebus apella*). Anim. Cogn..

[17] Murphy BL, Arnsten AF, Jentsch JD, Roth RH (1996). Dopamine and spatial working memory in rats and monkeys: Pharmacological reversal of stress-induced impairment. J. Neurosci..

[18] McEwen BS, Sapolsky RM (1995). Stress and cognitive function. Curr. Opin. Neurobiol..

[19] Dias-Ferreira E (2009). Chronic stress causes frontostriatal reorganization and affects decision-making. Science.

[20] Mattarella-Micke A, Mateo J, Kozak MN, Foster K, Beilock SL (2011). Choke or thrive? The relation between salivary cortisol and math performance depends on individual differences in working memory and math-anxiety. Emotion.

[21] Lautenbach F, Laborde S, Achtzehn S, Raab M (2014). Preliminary evidence of salivary cortisol predicting performance in a controlled setting. Psychoneuroendocrinology.

[22] Abreu CT (2006). A novel working memory test using capuchin monkey (*Cebus apella*) emotional faces. Neurobiologia.

[23] Webster MF, Brosnan SF (2021). The effects of positive and negative experiences on subsequent behavior and cognitive performance in capuchin monkeys (*Sapajus* [*Cebus*] *apella*). J. Comp. Psychol..

[24] Colares Leal TR, de Faria Brino AL, de Almeida Costa LA, de Faria Galvão O, McIlvane WJ (2020). Acquisition and maintenance of delayed matching-to-sample in tufted capuchin monkeys. J. Exp. Anal. Behav..

[25] Basile BM, Hampton RR (2013). Dissociation of active working memory and passive recognition in rhesus monkeys. Cognition.

[26] D’Amato M, Worsham RW (1972). Delayed matching in the capuchin monkey with brief sample durations. Learn. Motiv..

[27] Ter Horst JP, de Kloet ER, Schächinger H, Oitzl M (2012). Relevance of stress and female sex hormones for emotion and cognition. Cell. Mol. Neurobiol..

[28] Beiko J, Lander R, Hampson E, Boon F, Cain DP (2004). Contribution of sex differences in the acute stress response to sex differences in water maze performance in the rat. Behav. Brain Res..

[29] Dohmen TJ (2008). Do professionals choke under pressure?. J. Econ. Behav. Organ..

[30] Beilock SL, Kulp CA, Holt LE, Carr TH (2004). More on the fragility of performance: Choking under pressure in mathematical problem solving. J. Exp. Psychol. Gen..

[31] Oudejans RRD, Pijpers JR (2010). Training with mild anxiety may prevent choking under higher levels of anxiety. Psychol. Sport Exerc..

[32] Lupien SJ (2005). Stress hormones and human memory function across the lifespan. Psychoneuroendocrinology.

[33] Wheeler BC, Tiddi B, Kalbitzer U, Visalberghi E, Heistermann M (2013). Methodological considerations in the analysis of fecal glucocorticoid metabolites in tufted capuchins (*Cebus apella*). Int. J. Primatol..

[34] Rich EL, Romero LM (2005). Exposure to chronic stress downregulates corticosterone responses to acute stressors. Am. J. Physiol. Regul. Integr. Comp. Physiol..

[35] Lupien SJ, McEwen BS, Gunnar MR, Heim C (2009). Effects of stress throughout the lifespan on the brain, behaviour and cognition. Nat. Rev. Neurosci..

[36] Wang ZW, Shah P (2014). The effect of pressure on high- and low-working-memory students: An elaboration of the choking under pressure hypothesis. Br. J. Educ. Psychol..

[37] Sattizahn JR, Moser JS, Beilock SL (2016). A closer look at who “chokes under pressure”. J. Appl. Res. Mem. Cogn..

[38] Burling R (1993). Primate calls, human language, and nonverbal communication [and comments and reply]. Curr. Anthropol..

[39] Rawlings BS, Legare CH, Brosnan SF, Vale GL (2021). Leveling the playing field in studying cumulative cultural evolution: Conceptual and methodological advances in nonhuman animal research. J. Exp. Psychol. Anim. Learn. Cogn..

[40] Byrnit J (2006). Primate theory of mind: A state of the art review. J. Anthropol. Psychol..

[41] Roma PG (2007). Mark tests for mirror self-recognition in capuchin monkeys (*Cebus apella*) trained to touch marks. Am. J. Primatol. Off. J. Am. Soc. Primatol..

[42] Washburn DA, Rumbaugh DM (1992). Testing primates with joystick-based authomated apparatus—Lessons from the Language Research Center’s computerized test system. Behav. Res. Methods Instrum. Comput..

[43] Evans TA, Beran MJ, Chan B, Klein ED, Menzel CR (2008). An efficient computerized testing method for the capuchin monkey (*Cebus apella*): Adaptation of the LRC-CTS to a socially housed nonhuman primate species. Behav. Res. Methods.

[44] Pontecorvo MJ, Sahgal A, Steckler T (1996). Further developments in the measurement of working memory in rodents. Cogn. Brain Res..

[45] Evans TA, Perdue BM, Parrish AE, Beran MJ (2014). Working and waiting for better rewards: Self-control in two monkey species (*Cebus apella* and *Macaca mulatta*). Behav. Processes.

[46] Whitten PL, Stavisky R, Aureli F, Russell E (1998). Response of fecal cortisol to stress in captive chimpanzees (*Pan troglodytes*). Am. J. Primatol..

[47] Bates D, Maechler M, Bolker B, Walker S (2015). Fitting linear mixed-effects models using lme4. J. Stat. Softw..

[48] R Development Core Team (2016). R: A Language and Environment for Statistical Computing.

