# Supplementary material for: Endogenous cortisol correlates with performance under pressure on a working memory task in capuchin monkeys
Source: Sci Rep. 2022 Jan 19;12:953. doi: 10.1038/s41598-022-04986-6 (PMC8770687; doi:10.1038/s41598-022-04986-6)
Supplement: Supplementary file 1 — Supplementary Information. [file 41598_2022_4986_MOESM1_ESM.docx]

*Supplement: Analysis Including Extra Sessions*

Our *a priori* testing criterion was for all subjects to complete 15 sessions on the computerized task. Due to scheduling of testing time at the LRC, some subjects (*N* = 9) completed more than 15 sessions of the task (Range: 16-18). Because we had set a criterion of 15 sessions in our research plan, we chose to report these results as our main analysis. However, in the interest of transparency, we also present the same analyses done above with these added sessions included (*Table S1, Table S2*). The only difference between these supplemental models and our analyses reported above is that with additional data, session number and the interaction between the session number and average cortisol (log-transformed) were also significant predictors in the second model (*Table S2*). Average cortisol (log-transformed) remained a significant predictor in this model, as in our main text analyses. We suspect that the significant interaction effect in this model was driven by high performance on later sessions by the monkeys that completed these extra sessions, which, as discussed in the main text, are subject to experience effects in which practice under pressure improves performance under pressure.

*Table S1.* Linear mixed-model of proportion correct predicted by pressure condition and session number, with subject included as a random effect. For the categorical predictor of pressure condition, the intercept is “high-pressure”; for categorical predictor of sex, the intercept is “female”. Significant estimates and their *p*-values are bolded.

|  | **Proportion Correct** | | | |
| --- | --- | --- | --- | --- |
| *Predictors* | *Estimates* | *SE* | *Conf. Int. (95%)* | *p* |
| (Intercept) | **0.42** | 0.02 | 0.37 – 0.47 | **<0.001** |
| Condition | 0.00 | 0.01 | -0.02 – 0.02 | 0.773 |
| Session Number | **0.01** | 0.00 | 0.01 – 0.01 | **<0.001** |
| Sex | 0.06 | 0.04 | -0.01 – 0.14 | **0.086** |
| N _Subject_ | 20 | | | |
| Marginal R^2^ / Conditional R^2^ | 0.263 / 0.506 | | | |
|  |  | | | |

*Table S2*. Linear mixed-model of difference score predicted by average cortisol and session number, with subject included as a random effect. Significant estimates and their *p*-values are bolded.

|  | **Difference Scores** | | | |
| --- | --- | --- | --- | --- |
| *Coefficient* | *Estimates* | *SE* | *Conf. Int (95%)* | *p* |
| Intercept | **0.38** | 0.14 | 0.10 – 0.65 | **0.008** |
| Cortisol (log ng/g) | **-0.14** | 0.05 | -0.25 – -0.04 | **0.006** |
| Session Number | **-0.03** | 0.01 | -0.05 – 0.00 | **0.036** |
| Cortisol x Session Number | **0.01** | 0.00 | 0.00 – 0.02 | **0.024** |
| N _Subject_ | 19 | | | |
| Marginal R^2^ / Conditional R^2^ | 0.040 / 0.115 | | | |

*Supplement: Cortisol Assay Protocol and Validation Results*

A urine sample was serially diluted 1:2 with Assay Buffer and measured in the assay. The recovered concentration was determined from the standard curve and samples were linear within a range of 100% + 15% relative to the designated dilution. Observed values were not statistically different from expected values (Pearson *X^2^*(3) = 6.0, *p* = 0.11).

*Table S3*. Linearity Validation

| **Sample ID** | **Dilution Factor (DF)** | **Observed Concentration (ng/g)** | **Observed x DF (ng/g)** | **Expected (ng/g)** | **% Recovery** |
| --- | --- | --- | --- | --- | --- |
| 12 | 1 | 280.79 | 280.79 | 280.79 | - |
|  | 2 | 116.24 | 232.48 | 280.79 | 82.79 |
|  | 4 | 49.05 | 196.20 | 280.79 | 84.4 |
|  | 8 | 21.48 | 171.80 | 280.79 | 87.57 |
| **Average % Recovery** | | | | | **84.92** |

*Supplement: Results of Linear Models Assessing Demographic Features’ Effect on Cortisol*

In our sample set, none of the demographic factors that we assessed (social rank, age, or sex) were significantly correlated with baseline cortisol, as assessed by linear models (social rank, dominant as referent: *b* = 0.20, 95% CI = [-0.28, 0.68], *t* = 0.84, *p* = 0.41; age: *b* = -0.04, 95% CI = [-0.08, 0.01], *t* = -1.65, *p* = 0.11; sex, female as referent: *b* = -0.12, 95% CI = [-0.61, 0.37], *t* = -0.49, *p* = 0.63).

*Supplement: Additional Information About Capuchin Enclosures*

*Table S4.* Dimensions of Indoor and Outdoor Enclosures for Each Capuchin Group

| **Group** | **Number of Monkeys in Group** | **Available Space**  **(Indoor Enclosure)** | **Available Space**  **(Outdoor Enclosure)** | **Total Available Space** |
| --- | --- | --- | --- | --- |
| 1 | 6 | 52.18 m^3^ | 48.50 m^3^ | 100.68 m^3^ |
| 2a | 5 | 18.15 m^3^ | 33.09 m^3^ | 51.24 m^3^ |
| 2b | 4 | 18.15 m^3^ | 111.69 m^3^ | 129.84 m^3^ |
| 3 | 9 | 38.84 m^3^ | 444.02 m^3^ | 482.86 m^3^ |
| Lanson 1 | 4 | 26.14 m^3^ | 188.42 m^3^ | 214.56 m^3^ |
| Lanson 2 | 2 | 13.07 m^3^ | 53.82 m^3^ | 66.89 m^3^ |

**Supplementary Methods**

*Expanded Information about Subjects and Testing Setup*

Monkeys lived in one of five established mixed-sex social groups at the Language Research Center, each of which lived in their own large indoor/outdoor enclosure with extensive material enrichment and climbing structures. These social groups are stable and there is no invasive research at the LRC, suggesting that our monkeys’ baseline levels of stress are consistent with normal, everyday life in a typical capuchin. All monkeys received a species-appropriate diet of fruits and vegetables, as well as supplementary monkey chow and additional enrichment. Provisioning was not contingent on testing; therefore, subjects were never food- or water-deprived for testing purposes.

All subjects had at least one year of experience with the cognitive testing set up, including both the test boxes and the computerized testing system, prior to beginning the test. The computer task was programmed using Python 3.6 and run on a Windows XP operating system, except for one individual who completed the study on a Raspberry Pi system; the task design was identical between the Windows XP and Raspberry Pi programs.

*Details about Initial Round of Testing*

In an initial round of testing for eight of our subjects, we used more difficult abstract stimuli, but almost all monkeys dropped to chance level performance on both high-pressure trials and low-pressure trials (25% correct respectively). Their performance on even low-pressure trials never improved throughout multiple sessions (Session - Range: 2-8; Total trials – Range: 243-1179). Given that all of these monkeys had previously passed criterion (75% correct) in the training phase, this suggested that the stimuli were too hard to discriminate. Further, having a large number of potential stimuli introduced the possibility that monkeys could rely on simple familiarity with the most recent stimuli rather than working memory to solve the task ^31,39^. To reduce the ability to use this familiarity cue, and provide simpler stimuli, we developed the solid black symbols used in the test sessions and limited the number of stimuli to eight. We then presented this new set of stimuli to the monkeys in a new testing phase, with no additional training sessions; our analyzed dataset comprised the sessions from this testing phase. Although most monkeys suffered a small initial performance decrement when introduced to these new stimuli, all monkeys performed overall above chance in their first session, hence avoiding both floor and ceiling effects.
